# The Mouse Superior Colliculus as a Model System for Investigating Cell Type-Based Mechanisms of Visual Motor Transformation

**DOI:** 10.3389/fncir.2018.00059

**Published:** 2018-07-24

**Authors:** Ana F. Oliveira, Keisuke Yonehara

**Affiliations:** ^1^DANDRITE – Danish Research Institute of Translational Neuroscience, Nordic EMBL Partnership for Molecular Medicine, Aarhus, Denmark; ^2^Department of Biomedicine, Aarhus University, Aarhus, Denmark

**Keywords:** superior colliculus, visual processing, sensorimotor transformation, retinal ganglion cell, functional connectivity

## Abstract

The mouse superior colliculus (SC) is a laminar midbrain structure involved in processing and transforming multimodal sensory stimuli into ethologically relevant behaviors such as escape, defense, and orienting movements. The SC is unique in that the sensory (visual, auditory, and somatosensory) and motor maps are overlaid. In the mouse, the SC receives inputs from more retinal ganglion cells than any other visual area. This makes the mouse SC an ideal model system for understanding how visual signals processed by retinal circuits are used to mediate visually guided behaviors. This Perspective provides an overview of the current understanding of visual motor transformations operated by the mouse SC and discusses the challenges to be overcome when investigating the input–output relationships in single collicular cell types.

## Introduction

The superior colliculus (SC) is an evolutionarily conserved brain region found in mammals, homologous to the tectum in non-mammalian vertebrate species. It receives retinotopically organized synaptic inputs from retinal ganglion cells and reconstructs the spatial structure of the visual image (Cang and Feldheim, 2013; Cang et al., 2018). Historically, the SC has been studied in order to understand two different biological problems. The first is the mechanisms governing how topographic axonal projections from the chick and mouse retinas become established. These rely on axon guidance molecules (Frisén et al., 1998; Feldheim et al., 2000; Sweeney et al., 2015; Ito and Feldheim, 2018) and on spontaneous retinal waves (Chandrasekaran, 2005; Liu et al., 2014; Ito and Feldheim, 2018). However, until recently, the diversity of presynaptic retinal ganglion cell types (Kong et al., 2005; Völgyi et al., 2009; Sümbül et al., 2014; Baden et al., 2016) and postsynaptic collicular cell types (Mooney et al., 1988b; Gale and Murphy, 2014; Shang et al., 2015) has seldom been related to the organization of the retino-collicular projection (McIlwain, 1978; Hong et al., 2011; Joesch et al., 2016; Reinhard et al., 2018). The second problem concerns the neural mechanisms underlying saccadic eye movements in non-human primates (Schiller et al., 1980, 1979; Campos et al., 2006; Basso and May, 2017).

In rodents, the SC has also been used for studying innate behaviors related to avoidance or orientation (Sahibzada et al., 1986; Dean et al., 1988, 1989). These output behaviors are of great ecological value: the detection of (and consequent escape from) a predator, or effective localization and orientation adjustments to catch prey, can determine survival. Although these SC functions were uncovered several decades ago, it was not until recently that the physiological and behavioral roles of each collicular cell type began to be investigated (Gale and Murphy, 2014; Shang et al., 2015, 2018; Wei et al., 2015).

In the coming years, we expect we will determine a unified understanding of the function of the mouse SC at many levels: from gene function, cell types, and circuits to behavior. This Perspective aims to discuss the advantages of using the mouse SC as a model system for investigating the contribution of individual visual channels to visually guided behaviors, and proposes future research directions.

## Functional Organization of the Superficial Layers of the Mouse Superior Colliculus

The SC can be subdivided in the visuosensory and the motor layers. The latter consists of the intermediate and deeper layers. The superficial layers are visuosensory and include (from the surface): the *stratum zonale* (SZ), the *stratum griseum superficiale* (SGS), and the *stratum opticum* (SO; May, 2006; Ito and Feldheim, 2018).

In the mouse, the superficial layers of the SC (sSC) are the major retino-recipient structure in the brain, receiving input from ca. 90% of retinal ganglion cells (Ellis et al., 2016), and from the striate and extrastriate visual cortex (Wang and Burkhalter, 2013).

Visual responses to the appearance, disappearance, or movement of a stimulus were first detected in the mouse sSC several decades ago (Dräger and Hubel, 1975a,b). Later, single-unit extracellular recordings from the sSC in anesthetized mice during visual stimulation revealed several types of visual responses, including ON/OFF responses to flashing spot stimuli and orientation-selective (OS) responses. Interestingly, there were no changes in OS responses following a V1 lesion or dark-rearing-mediated visual deprivation (Wang et al., 2010), suggesting that collicular OS responses might either emerge *de novo* in the SC or be inherited from the retina. In addition, *in vivo* two-photon calcium imaging recordings identified direction-selective (DS) neurons in the most superficial lamina of the SC, the density of which declines with increasing distance from the surface of the SC (Inayat et al., 2015). Recently, it has been reported that DS responses in the sSC are inherited from the retina (Shi et al., 2017).

## The SC as a Model System for Visual Processing

Visual processing begins in the retina, where ca. 40 types of ganglion cells have been identified (Baden et al., 2016). Evidence from zebrafish (Robles et al., 2014) and mice (Ellis et al., 2016) indicates that there is massive divergence and convergence of axonal projections from retinal ganglion cell types to the brain. In other words, many ganglion cells project to multiple brain targets using collaterals (Ellis et al., 2016; Huang et al., 2017), and single brain centers receive inputs from multiple retinal ganglion cell types (Ellis et al., 2016; Reinhard et al., 2018). However, it is not yet understood how different ganglion cell types contribute to animal behavior, except for a few specialized cell types such as melanopsin-positive ganglion cells (Chen et al., 2011; Schmidt et al., 2011) or ON DS cells (Yonehara et al., 2009; Dhande et al., 2013).

The retina can be viewed as a parallel assemblage of small circuit modules represented by approximately 40 mosaics of retinal ganglion cells. Is the SC also functionally organized in parallel modules, operating the same computation throughout the SC? Recently, a column-like organization of OS cells was identified in the mouse SC where all angles and positions are not covered uniformly in the sSC. Feinberg and Meister (2015) revealed large patches containing OS cells with similar tuning. Ahmadlou and Heimel (2015) reported that neurons in the same column tend to prefer the same orientation, which is parallel to the concentric circle around the center of the visual field; this spatial organization could allow SC neurons to best respond to an expanding and receding optic flow. Another example of non-uniform coverage is the clustered distribution of the axon terminals of a transient OFF alpha ganglion cell type (Huberman et al., 2008) and an ON–OFF DS ganglion cell type (Rivlin-Etzion et al., 2011) along the surface of the SC, failing to cover all retinotopic locations on the SC. Investigating the synaptic and circuit mechanisms underlying the tuning to the expanding and receding optic flow in the sSC could reveal the key principles governing retino-collicular visual processing.

## The SC Mediates Tractable Behaviors

The rodent SC has commonly been associated with three types of output: escape/freezing defense-like behaviors, orienting movements, and autonomic responses. Defense-like behaviors consist of movements directed away from aversive stimuli, whereas orienting movements are generally directed toward attractive stimuli (Dean et al., 1989). Autonomic responses include marked changes in heart rate and blood pressure, and cortical arousal in response to visual emergencies (Redgrave and Dean, 1985; Keay et al., 1988).

Investigations into avoidance behaviors after visual stimulation have demonstrated that mice freeze and/or escape in response to a looming stimulus in the upper visual field, but not in the lower visual field, thereby suggesting that behavioral decisions are made based on the location of the stimulus within the visual field (Yilmaz and Meister, 2013; DeFranceschi et al., 2016). Follow-up studies have revealed that the sSC play a role in this behavior (Shang et al., 2015, 2018; Wei et al., 2015; Huang et al., 2017).

Orienting movements performed by the mouse are also being investigated. Mice exposed to crickets exhibit robust prey capture behavior and this behavior relies on vision (Hoy et al., 2016). While it has not yet been confirmed that the SC has a role in prey capture behavior in the mouse, undercutting the SC in the hamster impaired the pursuit of crickets (Finlay et al., 1980). Similarly, the SC has been shown to be involved in prey capture in other vertebrates (Ewert, 1974; Semmelhack et al., 2014).

## The SC as a Model System for Sensorimotor Transformation

The SC processes both aversive and appetitive visual stimuli, generating motor output responses related to avoidance and orientation, but the exact contributions of different retinal ganglion cell types to these visual motor transformations are not yet understood.

It has been suggested that one type of OFF ganglion cell in the retina is approach sensitive, as it responds to expanding, but not receding, black spots (Münch et al., 2009). Another population of ganglion cells, characterized as having the smallest and densest receptive fields, is thought to serve as an alarm neuron for overhead predators (Zhang Y. et al., 2012). How these approach or alarm retinal signals are processed by the sSC circuitry and transmitted to downstream premotor areas remains unknown.

Interestingly, stimulating the medial SC evokes avoidance or defense reactions in rodents, whereas stimulating the lateral SC elicits orienting or approach responses (Sahibzada et al., 1986; Dean et al., 1988). Because the medial and lateral SCs analyze the upper and lower visual field, respectively, these findings echo the behavioral observations that looming stimuli evoke escape/freeze behaviors only when presented from above.

**FIGURE 1:**
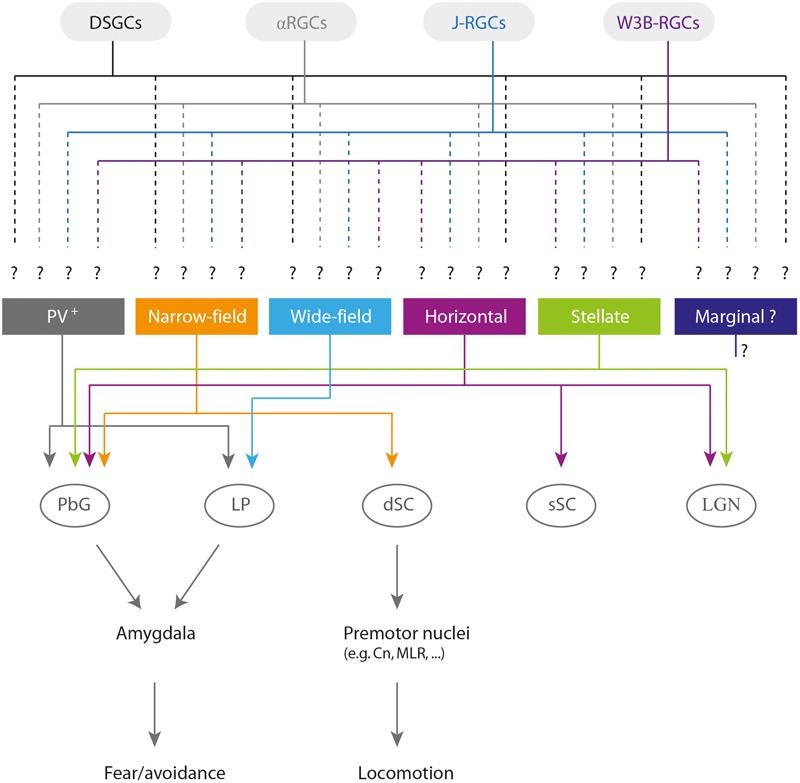
Overview of the sSC cell types (Gale and Murphy, 2014; Inayat et al., 2015; Shang et al., 2015, 2018), potential inputs from retinal ganglion cell types (Sanes and Masland, 2015), relevant output brain targets, and behavioral roles. Note that the identity of the retinal ganglion cells projecting to each collicular cell type remains unknown. Abbreviations: DSGCs, direction-selective ganglion cells; αRGCs, alpha retinal ganglion cells; J-RGCs, JAM-B-expressing OFF retinal ganglion cells; PV^+^, parvalbumin-positive neurons; dSC, deep layers of the superior colliculus; PbG, parabigeminal nucleus; LP, lateral posterior nucleus of the thalamus; sSC, superficial layers of the superior colliculus; LGN, lateral geniculate nucleus of the thalamus; Cn, cuneiform nucleus; MLR, mesencephalic locomotor region.

Neurons in the sSC project to the intermediate (iSC) and deep layers (dSC) of the SC (Mooney et al., 1988a). Projections from deeper layers to the nuclei of the brainstem can be either contralateral (from the lateral regions of the SC) or ipsilateral (from medial regions; Bickford and Hall, 1989). Consequently, contralateral projections tend to mediate orienting behaviors, while ipsilateral projections tend to mediate avoidance behaviors (Redgrave et al., 1996a,b; Comoli et al., 2012). In line with these findings, stimulating the cuneiform nucleus (Cn) and the parabigeminal nucleus (PbG), two of the main targets of ipsilateral descending projections (Redgrave et al., 1987), evokes escape/freezing behaviors in rodents (Parker and Sinnamon, 1983; Mitchell et al., 1988; Shang et al., 2015; Caggiano et al., 2018). Furthermore, the Cn receives projections from the medial part of the SC, a region representing the upper visual field (Westby et al., 1990). Future research should examine how distinct dSC output cells collect information from retinal ganglion cell types *via* sSC neurons to extract salient features from the visual scene (**Figure 1**).

Together, these reports suggest that visual pathways dedicated to survival-related behaviors are hard-wired by segregated neuronal projections, possibly by intrinsic genetic mechanisms.

## Genetic Labeling of SC Cell Types in Mice

To understand the circuit mechanisms underlying visual processing and sensorimotor transformation, cell type-based studies are crucial, as they make it possible to link light responses, connectivity, behavior, and gene expression (**Figure 1**). As with zebrafish (Robles et al., 2011), gaining genetic access to cell types in the mouse SC will be critical for untangling the functional connectivity of neuronal circuits in the SC.

To date, four distinct cell types have been identified in the mouse sSC: narrow-field, wide-field, horizontal, and stellate cells. It has been suggested that a group of small cells at the border of the SZ, with dendrites extending toward the upper SGS, make up a fifth cell type, the marginal cells (May, 2006). However, attempts to characterize the electrophysiological properties of this cell type have failed to distinguish it from stellate cells (Gale and Murphy, 2014). Nonetheless, a population of DS cells with compact receptive fields, containing both excitatory and inhibitory neurons, has been found in the superficial SGS: these could be marginal cells (Inayat et al., 2015).

Narrow-field cells are labeled with Cre recombinase in the transgenic mouse Grp-KH288-Cre (Gerfen et al., 2013; Gale and Murphy, 2014). They are small, with thick dendrites extending dorsally toward the SC surface and ventrally toward the SC deeper layers. They have small receptive fields, respond to slowly moving stimuli and are DS (Gale and Murphy, 2014). Narrow-field cells project to the deeper layers of the SC and to the PbG (**Figure 1**). Given their projection pattern and their physiological responses, it is tempting to speculate that these cells could be involved in signaling the location of salient visual inputs to the iSC and therefore shifting the gaze toward a target, and/or in avoidance responses mediated by the SC–PbG–amygdala pathway (Shang et al., 2015).

Wide-field cells are labeled with Cre recombinase in the transgenic mouse Ntsr1-GN209-Cre (Gerfen et al., 2013; Gale and Murphy, 2014). These cells display dendrites extending diagonally to the surface of the SC and forming a large field. They respond best to slowly moving stimuli and can be DS and/or OS. These cells project to the lateral posterior nucleus of the thalamus (LP; **Figure 1**; Gale and Murphy, 2014), which makes them a good candidate to mediate avoidance behaviors *via* the pathway connecting the SC–LP–amygdala (Wei et al., 2015).

Horizontal cells are labeled with Cre recombinase in the transgenic mouse line GAD2-Cre (Gerfen et al., 2013; Gale and Murphy, 2014). These cells have large receptive fields, respond best to either large stationary or fast-moving visual stimuli, and are rarely DS. They provide inhibitory input to both the dorsal and ventral lateral geniculate nucleus of the thalamus (LGN) and to the PbG (**Figure 1**; Gale and Murphy, 2014).

Stellate cells have multiple dendrites with no clear orientation and have small receptive fields. They respond best to small visual stimuli and project to both the PbG and LGN (**Figure 1**; Gale and Murphy, 2014, 2018). These cells are labelled in the transgenic mouse line Rorb-Cre, but horizontal and narrow-field cells are also labelled in this mouse line (Gale and Murphy, 2018), thereby hindering the investigation of the specific role of stellate cells in innate visual motor behavior.

The transgenic mouse line PV-ires-Cre labels cells in the SGS and SO known to project to the amygdala *via* the PbG and to mediate escape and freezing behavior (**Figure 1**; Shang et al., 2015, 2018). Recently, it has been shown that a subpopulation of parvalbumin-positive (PV^+^) cells located in the SO projects to the LP and specifically mediates freezing behavior (**Figure 1**; Shang et al., 2018). Even though it was demonstrated that the PV^+^ neurons in these studies were glutamatergic (Shang et al., 2015, 2018), PV^+^ cells in the SC form a distinct mixed population of glutamatergic and GABAergic neurons with heterogeneous morphological and electrophysiological properties (Villalobos et al., 2018). While the morphological analysis described by Shang et al. (2015) suggests that these neurons might be narrow-field cells, a new report demonstrated that PV^+^ neurons in the sSC also include stellate and horizontal cells (Villalobos et al., 2018). The diversity encountered among PV^+^ neurons could indicate that these neurons serve multiple circuit and behavioral functions.

## Future Directions and Challenges: Visual Motor Transformation at the Level of a Single Cell Type

A plethora of new molecular, genetic, and imaging tools that has become available in recent years now means that visual motor transformations can be dissected at the single-cell type level. These tools make it possible to identify the locus of synapses within specific neuronal circuits mediating visual motor integration; this information will enable molecular and activity-dependent mechanisms underlying the circuit assembly to be studied.

Advances in mouse genetics have provided most of the essential tools for exploring the role of single cell types in visual motor behavior. Multiple mouse lines are now available, expressing Cre recombinase in specific cell types of particular brain regions (Taniguchi et al., 2011; Gerfen et al., 2013). However, a systematic approach for identifying and labeling SC cell types has rarely been applied (Byun et al., 2016). An unbiased method for characterizing cell types based on gene expression pattern, such as dropSeq (Klein et al., 2015; Macosko et al., 2015), could be used to identify cell types based on specific molecular markers. These could, then, be exploited to create transgenic mice in which a single cell type is labeled with Cre recombinase. Other approaches for targeting individual cell types could be selecting AAVs with tropism and/or a promoter for selective neuronal populations (Dimidschstein et al., 2016), or nanobodies that are reconstituted only when presented with a specific antigen (Tang et al., 2013).

Having a valuable collection of mouse lines with labeled SC cell types will provide excellent opportunities for linking their activity and connectivity. It will then be possible to examine how the convergence of ganglion cell types is organized at the level of brain targets’ single cell types (**Figure 1**; Rompani et al., 2017) by combining trans-synaptic tracing with modified viral tracers expressing activity sensors and two-photon imaging (Yonehara et al., 2013; Wertz et al., 2015; Zingg et al., 2017).

Another unanswered question is how retinal signals processed by the sSC circuitry are transmitted to downstream premotor areas (**Figure 1**). Recent work has examined a similar problem using retrograde rabies virus-based trans-synaptic circuit tracing, and determined the combinations of neuronal pathways originating from retinal ganglion cell types projecting to two brain centers that mediate avoidance responses *via* the sSC (Reinhard et al., 2018). Follow-up experiments using specific inactivation and activation of the involved retinal ganglion cell types will be fundamental to understanding the contribution of each ganglion cell type to visual motor transformations.

Next, it will be imperative to examine how the retino-collicular connectivity that underlies visual processing operated by individual genetically labeled SC cell types is established by genetic- and activity-dependent mechanisms. The genetic mechanisms can be analyzed by testing the effect of gene knockdown in genetically labelled presynaptic ganglion cell types or postsynaptic sSC cell types, using conditional knockout mice or adeno-associated virus- or electroporation-mediated cell type-specific delivery of RNAi or CRISPR/Cas9 constructs. The contribution of activity-dependent mechanisms can be addressed by transiently activating or suppressing presynaptic or postsynaptic activity in a cell type- and developmental-specific manner, using optogenetic and chemogenetic tools (Zhang J. et al., 2012).

Finally, understanding to what extent the mechanistic insights obtained from the mouse SC are conserved across different animal species will be fundamental. Such work will deepen our understanding of how the species-specific functional organization of the SC is built to meet ethological requirements.

## Conclusion

Here, we propose the mouse SC as an outstanding model for investigating sensorimotor transformation at the single-cell level. First, the laminar organization of the mouse (May, 2006) facilitates the identification of individual cell types. Second, the mouse is a genetically tractable animal and individual cell types can be labelled with DNA recombinase (Gerfen et al., 2013; Gale and Murphy, 2014), enabling manipulation and monitoring of specific collicular cell types. Third, the sSC receives monosynaptic inputs from retinal ganglion cells (Ellis et al., 2016) and is located relatively superficially, being accessible for imaging with two-photon microscopy (Ahmadlou and Heimel, 2015; Feinberg and Meister, 2015). Fourth, in the mouse, ca. 90% of the retinal ganglion cells project to the SC. Fifth, several behavioral paradigms are available for probing visual motor transformations processed by the SC (Shang et al., 2015; Wei et al., 2015). Last, breeding mice is faster, cheaper, and easier than breeding non-human primates, making the mouse a readily available tool to the wider scientific community. For these reasons, we expect the mouse SC to become heavily studied in the next years as a valuable system for examining cell type-based mechanisms underlying visual motor processing.

## Author Contributions

AO and KY wrote, edited, and revised the manuscript.

## Conflict of Interest Statement

The authors declare that the research was conducted in the absence of any commercial or financial relationships that could be construed as a potential conflict of interest.
